# Neoliberal Economic Policies’ Effects on Perceptions of Social Justice and Sociopolitical Participation in Portugal

**DOI:** 10.3389/fpsyg.2021.694270

**Published:** 2021-11-29

**Authors:** Mariana Lucas Casanova, Patrício Costa, Rebecca Lawthom, Joaquim L. Coimbra

**Affiliations:** ^1^Faculty of Psychology and Education Sciences of the University of Porto, Porto, Portugal; ^2^Life and Health Sciences Research Institute (ICVS), School of Medicine, University of Minho, Braga, Portugal; ^3^ICVS/3B’s – PT Government Associate Laboratory, Braga/Guimarães, Portugal; ^4^Faculty of Social Sciences, School of Education, The University of Sheffield, Sheffield, United Kingdom

**Keywords:** neoliberalism, psychosocial uncertainty, social justice, sociopolitical control, social dominance orientation (SDO), financial deprivation, agency, structural equation modelling (SEM)

## Abstract

Contemporary societies challenge long-standing projects of the “good society” and social equality through neoliberal economic policies. Social forms of uncertainty generated by financial deprivation, precarity, and inequality seem to have effects on agency and coping and so socioeconomic and psychological consequences. This study aims to test these relationships, as well as a hypothesis on the potential impact of these constructs on beliefs of sociopolitical control and social dominance, which have implications for social justice. A mediation model explores the effects of financial access (the manifest benefit of work) on psychosocial uncertainty (which reflects the perception of uncertainty in the social context and the experience of its consequences within work, relationships, and the adoption of self-defeating beliefs) and on emotional coping strategies towards uncertainty, and their effects on personal agency, sociopolitical control (SPC), and social dominance orientation (SDO). Data are derived from a study of 633 participants in Portugal. Although personal agency is influenced by financial access and psychosocial uncertainty, it is not proved as a significant mediator for SPC and SDO. Nevertheless, financial access, psychosocial uncertainty, and emotional coping significantly contribute to the model, supporting the hypothesis that financial access protects against psychosocial uncertainty. Both have an impact on SPC and SDO. Therefore, financial deprivation and psychosocial uncertainty potentially contribute to extremism and populism in societies characterised by socially created forms of uncertainty. Implications of results for psychological intervention, namely in vocational/professional counselling, are discussed.

## Introduction

Uncertainty has become a central concern in contemporary Western societies. Throughout history, the development by science and industry of the ability to control (or manage) the challenges to human life created by nature led to a shift from a concern with naturally created uncertainties to socially created ones (Beck, 1992). Indeed, human attempts of controlling natural uncertainties have created new ones (e.g., climate change, social and economic inequalities, and pandemics). Contemporary societies challenge long-standing projects of the “good society” and social equality through neoliberal economic policies, which have engendered individualistic values, competitiveness, distrust, and individualisation, negating the role of ethical values, thus complicating individual coping strategies (Marcuse, 1964/1991; Prilleltensky, 1994; Bauman, 2001; Stiegler, 2004/2011; Coimbra and Menezes, 2009; Prilleltensky and Stead, 2012). So, these circumstances have both socioeconomic and psychological consequences, such as unemployment and precarity, jeopardizing well-being. Therefore, people face socially created forms of precarity and uncertainty (in work, relationships, communities) that create feelings of anxiety, fear, anger, alienation, and anomie (Standing, 2011; Lucas Casanova et al., 2019a). To those socially vulnerable, meaning and agency are compromised, and their powerlessness has political effects, undermining social trust (Fryer, 1992, 1998; Marris, 1996; Standing, 2011). These feelings go beyond individual psychological experiences and affect collective, social experiences, thus potentially contributing to an increase in extreme, populist, or conservative political groups, limiting solidarity and thus social justice (Marcuse, 1964/1991; Prilleltensky, 1994; Stiegler, 2004/2011). Macrosocial circumstances contribute to these phenomena. Portugal was one of the countries that were hit the hardest after the financial crisis of 2007/2008. The country underwent an economic crisis: unemployment rates and precarity levels increased, while austerity measures led to an erosion of welfare policies and a decrease in social benefits. Literature results report greater inequality in the country as a result of the crisis and austerity, compromising physical and mental health.

Located in Portugal, this study explores potential consequences of inequality, here assessed through financial access and psychosocial uncertainty (reflecting the individual – micro level – experience of macrosocial effects). Being a cross-sectional study, its results cannot be attributed to the consequences of the crisis in the country and the macrosocial circumstances it created (namely, liberalisation of labour laws). Nevertheless, the use of these individual variables may reflect these socioeconomic and political circumstances. Thus, the study tests a theoretical model of the relationship between financial access; psychosocial uncertainty (the interaction between the perception of uncertainty in the social context and its psychological experience) (Marris, 1996), which has effects on coping strategies towards uncertainty, mostly in terms of emotional coping (Lucas Casanova et al., 2021); with personal agency (Fryer, 1992, 1998); beliefs of sociopolitical control (SPC) (studied as the intrapersonal component of psychological empowerment – Zimmerman and Zahniser, 1991; Zimmerman, 1995; Peterson et al., 2006, 2011); and their potential effects on beliefs of social dominance orientation (SDO) and social inequality as political strategies, which may allow an analysis of meso level effects (Sidanius and Pratto, 1999; Pratto et al., 2006) — a hypothesis that, to the best of our knowledge, has not yet been tested.

The data used in this study were collected in Portugal in 2017/2018, a period in which the country was recovering from years of economic crisis due to the financial crisis of 2007/2008. We will explore how socioeconomic conditions of inequality and deprivation may negatively impact on well-being (through a focus on coping strategies towards uncertainty) but also undermine crucial psychological antecedents of citizenship and sociopolitical participation, as personal agency and SPC. A possible paradoxical effect of deprivation, inequality, and uncertainty on social dominance and anti-egalitarianism beliefs will also be explored.

### Financial Access, Psychosocial Uncertainty, Emotional Coping With Uncertainty and Personal Agency

Psychosocial uncertainty as a construct reflects the articulation between the perception of uncertainty within the social context and its psychological experience. It was developed to reflect and empirically test Marris’s thesis that there are social origins of uncertainty in contemporary Western societies: that uncertainty and the power to cope with it are unequally distributed – politics of uncertainty (Marris, 1996). The author proposes vulnerable people are led to adopt self-defeating strategies of coping with uncertainty by dominant people and groups through social discourses and policies that push uncertainty onto the powerless. The scale is composed of three dimensions: psychological consequences of uncertainty within work (concerns with work perceived as a consequence of uncertainty in the social context), relationships and community living (experiences of community deficit or distrust, perceived as a consequence of uncertainty), and self-defeating beliefs in coping with uncertainty (beliefs in not being able to cope with uncertainty). Based on this, the Psychosocial Uncertainty Scale (PS-US) was developed (Lucas Casanova et al., 2021). Results show women and workers (compared to students) experience more psychosocial uncertainty and its psychological consequences within work, as well as self-defeating beliefs in the possibility of coping with uncertainty. Moreover, the participants from lower sociocultural levels experience more psychosocial uncertainty and its psychological consequences within work, relationships and community living, and self-defeating beliefs in coping with uncertainty (Lucas Casanova et al., 2021).

Furthermore, a structural equation model shows that psychosocial uncertainty mediates the relationship between the access to manifest and latent benefits and emotional coping strategies of work towards uncertainty. The model explains emotional coping strategies by 70%, which endorses the existence of socioeconomic origins of uncertainty that may foster inequality and an interpretation of emotional coping strategies as self-defeating strategies that are a consequence of socioeconomic circumstances (Lucas Casanova et al., 2019a, b, 2021; Lucas Casanova, 2021). Additionally, results showed unemployed people were more challenged than permanent workers and that psychosocial consequences of uncertainty within work changed across time, demonstrating the macrosocial impact of the crisis in Portugal. The present study explores the potential effects of financial access (the manifest benefit of work) (Fryer, 1998), psychosocial uncertainty and emotional coping strategies towards uncertainty on personal agency, SPC and SDO. Financial access is assessed through this dimension from the Latent and Manifest Benefits Scale – LAMB – Scale (Muller et al., 2005; Sousa-Ribeiro, 2013). Emotional coping strategies are assessed using this dimension from the Uncertainty Response Scale (URS, Greco and Roger, 2001; Lucas Casanova et al., 2019b).

Regarding personal agency, it is expected to mediate the relationship between psychosocial uncertainty and emotional coping, and SPC and SDO. The personal agency scale was developed for this study to reflect the perspective of Fryer (1992, 1998) of personal agency as constrained by financial access. Given the effect of financial access on psychosocial uncertainty and emotional coping previously observed (Lucas Casanova, 2021), and its expected relationship with personal agency, financial access was defined as an exogenous variable in the present study. The literature suggests that financial deprivation undermines action, which subsequently affects feelings of agency and empowerment (Fryer, 1992, 1998). So, we hypothesise that psychosocial uncertainty may also predict personal agency (since uncertainty creates feelings of lack of control, undermining connections with the future). Additionally, we will test if both these variables (psychosocial uncertainty and agency) influence SPC and SDO, considering the relationship of agency with psychological empowerment (Fryer, 1992; Zimmerman, 1995).

### Sociopolitical Control

The concept of SPC represents beliefs in skills and abilities of people to influence sociopolitical systems, reflecting one of the components of psychological empowerment, the intrapersonal one (with cognitive and motivational components), focused on the public space (Zimmerman and Zahniser, 1991; Zimmerman, 1995). The interactional component involves critical awareness of individuals of resources necessary to achieve aims and understanding these environments, and the behavioural one refers to the actions that may affect desired outcomes (Zimmerman, 1995). Zimmerman and Zahniser (1991) developed the SPC Scale (SPCS), which is used in this study and is composed of two dimensions: leadership competence, which reflects self-perceptions of the ability to organise groups of people, and policy control, which reflects expectations and self-perceptions of the ability to influence policy decisions. Research has shown that educational levels have significant effects on both dimensions of SPCS; SPC is related with measures of alienation and community involvement; empowerment plays an important protective role in community quality of life and health, namely in the workplace, where socioeconomic status and empowerment contribute to health outcomes; high levels of policy control are more important for issues as school participation and perceived school importance than high levels of leadership competence (e.g., Zimmerman and Rappaport, 1988; Zimmerman and Zahniser, 1991; Marmot and Siegrist, 2004; Syme, 2004; Peterson et al., 2006). Moreover, interesting empirical results show that people trust more in the government when they experience ignorance in respect to a threatening social issue, avoiding learning more about it, seemingly desiring to keep their faith in the government as a trustworthy institution (Shepherd and Kay, 2012). However, generalised trust and trust in government have decreased in some countries (Rontos and Roumeliotou, 2013; Freire, 2016; OECD, 2020), and, so, it could be possible that this “blind trust” that reinforces ignorance may be transported to other social institutions that, nevertheless, also maintain the status quo, such as the media and social media.

Thus, SPC is a crucial concept to assess the psychological risks of high environmental demands, when individuals experience low levels of control over the environment – low psychological empowerment (Zimmerman and Zahniser, 1991; Peterson et al., 2006). Therefore, considering a social context characterised by uncertainty, high unemployment and/or precarity levels (which create financial deprivation and endanger a sense of personal agency), it is hypothesised that psychosocial uncertainty, emotional coping, and personal agency may significantly relate to the dimensions of SPCS. Moreover, SPC may predict beliefs in SDO and egalitarianism.

It is worth mentioning that neoliberal discourses have co-opted the concept of empowerment to signify individual, psychological empowerment, ultimately making individuals responsible for their failures through processes of responsabilization (Rutherford, 2018). Here, we are using the concept of psychological empowerment and SPC, considering its usefulness to capture individual experiences of SPC. However, it is crucial to emphasise that, despite it reflecting an individual, psychological experience, it does not mean that individuals are to be held accountable for their levels of empowerment in societies that consistently disempower them. As Zimmerman and Zahniser (1991) mention “high scores on the SPCS among disenfranchised individuals may portend health and mental health problems, because people may become frustrated in a world where they feel they have some control but are actually quite powerless.” (p. 201).

### Social Dominance Orientation

Social dominance theory (Sidanius and Pratto, 1999; Pratto et al., 2006) integrates ideas from authoritarian personality theory and cultural theories of ideology (Adorno et al., 1950), Marxism (Marx and Engels, 1845–1846), feminist anthropological analyses of family and labour, among others, to provide multiple levels of analysis of this concept. It attempts to understand and explain how group-based social hierarchies in which some group(s) that have greater social status and power enjoy more positive social value (material and symbolic resources as political power, wealth, protection, leisure, and education). In contrast, subordinate groups are left with deprived housing, unemployment or underemployment, stigmatisation, and poor living conditions in general. It considers three structures of hierarchy (age, gender, and arbitrary-set systems). The latter reflects social distinctions related to power as nationality, race, class, or religion since it seems to have only emerged in societies where there is economic surplus. Dominant social groups use hierarchy-enhancing (HE) legitimizing myths to retain their power, justify oppression and inequality, potentially without the need for violence. Individualistic values, meritocracy and the Protestant work ethic, are examples of such HE-legitimizing myths related to the existence of forms of uncertainty that are socially created (Lucas Casanova et al., 2019a). Alternatively, hierarchy-attenuating (HA)-legitimizing myths, such as social democracy, socialism, communism or human rights doctrines, seek to counter the dominance of specific social groups. The power of a legitimizing myth depends on adherence of subordinate groups to it, leading to ideological consensus across groups when subordinate groups acquiesce to it. Work and the institutions people work in are also relevant for this process, since the labour market and institutions reproduce HE or HA myths. Social dominance theory and the measurement instruments it has originated have proved to be extremely useful for understanding social processes related to social domination and inequality, relating SDO to right-wing authoritarianism (RWA), anti-immigration policies, racism or anti-equality policies, among many other crucial sociopolitical issues (e.g., Pratto et al., 1994; Sidanius and Pratto, 1999; Ho et al., 2015). Altemeyer (1998) suggested that SDO can account for a dominant expression of authoritarianism, while RWA would be its submissive expression. Additionally, results suggest that intolerance of ambiguity affects political conservatism and SDO (de Rojas, 2012) and that emotion avoidance has a positive association with SDO (Leone and Chirumbolo, 2008). Furthermore, research has demonstrated that uncertainty tolerance and intolerance of ambiguity predict political conservatism (Jost et al., 2003) and that increased uncertainty avoidance is related to weaker support for multicultural policies (Leong, 2008). Also, empirical results show that subjective perceptions of threat (perceptions of the world as dangerous or a “competitive jungle”) are associated with SDO (Jost et al., 2017). All these results support a possible relationship between emotional coping with uncertainty and SDO. Besides, results show that people with higher SDO levels show higher alienation levels and lower perceived control over sociopolitical issues (Nicol, 2007) and that SDO correlates negatively with tolerance of uncertainty (Nicol, 2009).

Theoretical analyses and empirical results suggest that uncertainty (as social instability) can drive sociopolitical and ideological extremism (in which the search for powerful autocratic leaders works as a way to provide some sense of security or certainty), or generate political violence (Hogg and Adelman, 2013; Hogg et al., 2013; Hogg, 2014; Wagoner and Hogg, 2017; Gøtzsche-Astrup, 2019, 2020; Stewart et al., 2019). However, the interpretation of empirical results depends on the definition of uncertainty being used (as personal insecurity, self-uncertainty, need for cognitive closure, intolerance of uncertainty or intolerance of ambiguity, perceived threat, or social uncertainty) and how it is measured. Shaffer and Duckitt (2013) propose that the most important effect of threat on RWA and SDO may not be intrapersonal forms but social, collective threats, in contrast to Adorno et al. (1950), who considered authoritarianism to be caused by personal insecurity and fears. Indeed, Onraet et al. (2013) found that external social threats were related to SDO and that controlling for them eliminated effects for internal, personal threats, which suggests that external uncertainty may be more significant for developing right-wing attitudes. However, Shaffer and Duckitt (2013) only found that external, social threat affected RWA (mostly threats to the ingroup), but not on SDO, suggesting they may have different psychological bases. Still, this study intends to explore if psychosocial uncertainty (which provides an assessment of the interaction between uncertainty generated in the social context and its psychological experience) may influence SDO levels since it may account for external, social forms of threat.

We diverge in two interpretations from SD theory: the evolutionary take of the theory on the invariance hypothesis (gender differences in which women have consistently shown lower levels of SDO), which seems to naturalise gender experiences; and the relative stability of SDO across time as evidence of it being a psychological trait (Pratto et al., 2006). Even though this study does not focus on these issues, this is relevant since we adopt a social-constructionist, developmental, ecological understanding of these issues (Gergen, 1996; Marris, 1996; Bronfenbrenner, 2005). Aligned with this, we question the interpretation of SDO as a trait and propose that, albeit part of a psychological structure (needing more time to change), it is a result of a set of life-long developmental experiences within the microsystem, mesosystem, exosystem, chronosystem, and macrosystem of an individual, thus being potentially sensitive to historical and social circumstances. Considering research results on correlates of SDO within personality, we believe that the situational influences that were used, for example, in experimental research with SDO, demonstrate the possibility of its change according to specific conditions (Levin, 1996 cite in Pratto et al., 1994; Sidanius and Pratto, 1999; Pratto et al., 2006; Ho et al., 2015). Indeed, empirical results suggest that there is a tendency for conservative shifts to the political right or towards more authoritarian institutions (e.g., conversion rates to authoritarian churches; increases in right-wing populism after the attacks of September 11, 2001, including in Portugal) in the face of historical and socioeconomic circumstances of crisis or instability in society (Jost et al., 2003). These results show that instability and uncertainty in the social context have an important role in these issues, probably more so than internal forms of uncertainty that consider it a psychological trait and neglect its relationship with the environment. This does not mean negating results that relate RWA to intolerance of uncertainty, or that conservatives show discomfort with job insecurity and wish to escape ambiguity, novelty, and uncertainty (Jost et al., 2003). However, social discourses of dominant groups preach on the need to “step outside your comfort zone” to adapt to change and be flexible, pushing uncertainty towards the ones who are powerless to escape it. Therefore, some groups have the power to elude uncertainty. In contrast, others do not, which means it may be more important for research on hierarchy and oppression to explore potential effects of social uncertainty on SDO and RWA.

In Western societies, neoliberal policies and discourses that reproduce individualistic, meritocratic values, and the protestant work ethic (which value discipline and sacrifice, as a justification to make subordinate groups responsible for their failures, thus turning failure into evidence of moral or psychological incompetence) are hegemonic (Prilleltensky, 1994; Chernomas and Hudson, 2009, 2010; Ho et al., 2015). So, inequality becomes a form of social control through the internalisation of these ideologies by subordinate groups. Politics of uncertainty create more uncertainty for subordinate groups while disempowering and depriving them of the strategies to cope with it by characterising the world as a zero-sum game in which power must be achieved at all costs (Lucas Casanova et al., 2019a).

Furthermore, different forms of anomie may lead to different emotions and different psychosocial outcomes. Tenhouten (2016) analysed these issues, proposing two forms of anomie: a passive, unintentional form that can occur as an outcome of unstable social conditions and personal experiences of uncertainty that generates introjected primary and secondary emotions, such as surprise, sadness, fear, disappointment, shame, and alarm, respectively, and may lead to unpremeditated homicide, suicide, discouragement, depression, and confusion; and an active, intentional form, based on self-interest (which is related to SDO), and that generates extrojected primary (anger, disgust, and joy) and secondary emotions (contempt, pride, and derisiveness), potentially leading to premeditated homicide, shamelessness, ruthlessness, immorality, and acquisitiveness. However, these two forms may be connected, and active anomie may contribute to the passive form of anomie, generating not only violence towards other groups but also having possible self-destructive effects, as self-defeating strategies to cope with uncertainty. Individuals may also suffer from both forms of anomie: “doubly anomic individual will live in fear of being treated with contempt or derision (attributes of an individual high in anomie 1), and filled with a sense of inferiority, together with grandiose fantasies about attaining impossible goals, will cling to an unstable ‘pride system”’ (Tenhouten, 2016, p. 481). On the other hand, this could allow a connection between RWA (as the submissive form of authoritarianism) and a passive form of anomie, while SDO (as the dominant expression of authoritarianism) would reflect an active form of anomie, which may help understand self-debilitation or false consciousness of subordinate groups.

One of the most important ways in which individuals interact with socioeconomic and political structures is through work, a context in which people often experience employment uncertainty, job insecurity, and precarity. Concerning this connection between vocational/professional realms, SPC and SDO, research has demonstrated a significant relationship between critical consciousness (assessed through the SDO scale and the SPCS) and progress in career development (Diemer and Blustein, 2006). Other studies have found a negative relationship between social generativity (related to inclusion and social equality attitudes) and SDO (Morselli and Passini, 2015). These findings support the relevance of the work context and the labour market in experiences of SPC and SDO and may suggest a possible interaction between external uncertainty and vocational/professional development (Blustein, 2019). So, it is expected that psychosocial uncertainty and the experience of its consequences within work and the labour market may contribute to SPC and SDO. On the other hand, work is how most people gain access to the financial benefits that allow “making a living” through wage labour.

Considering the anger and anomie that financial deprivation may cause, we expect financial access to be an exogenous variable that may influence the whole model, directly (potentially protecting from psychosocial uncertainty and emotional coping, increasing personal agency and SPC) or indirectly in the case of SDO, protecting from dominance orientation and anti-egalitarianism. Psychosocial uncertainty may contribute to a desire for group-based social dominance and inequality since it reflects HE legitimizing ideologies with impact on precarity and unemployment through policies in the area of work. This can also occur with people that are part of subordinate groups (through self-debilitation), which can also be perceived as a self-defeating strategy in the sense that people are acting against their group interest or acting in ways that reinforce stereotypes on their group, as self-fulfilling prophecies (Pratto et al., 2006). Nevertheless, it is also a reflection of psychological needs to reduce fear, anxiety, and uncertainty (Jost et al., 2003). Furthermore, Portugal (the context of the study) experienced in the last decade an economic crisis that created more psychosocial uncertainty, mainly at work, through unemployment and precarity (Lucas Casanova et al., 2019a; Lucas Casanova, 2021). Social inequality has increased in the country, as in other countries (Wilkinson and Pickett, 2009) and, for the first time since the 1974 revolution, in 2019, a far-right member of Parliament was elected. Therefore, this study intends to explore if a macrosocial climate of uncertainty may impact SDO, since increased inequality is related to increased SDO (Pratto et al., 2006), which will be assessed through financial access and psychosocial uncertainty. In this study, the SDO-7 short is used (Ho et al., 2015) to assess SDO in its dimensions – Pro/Anti Dominance and Pro/Anti Egalitarianism.

In summary, this study tests a structural equation model in which financial access is an independent variable with potential effects on PS-US dimensions, emotional coping, and personal agency (as potential mediators), SPC and SDO. It must be emphasised that these effects can only be fully assessed through longitudinal or comparative studies. Nonetheless, this study aims to test this theoretical model cross-sectionally, assessing the predictive power of these variables. So, the use of concepts as “effect” or “influence” throughout this article must be appreciated with this caveat in mind. Predicted effects were defined by theoretical orientations in the literature and a pilot study. Generally, it is hypothesised:

(1) Negative effects of financial access on psychosocial uncertainty (within work, relationships, and self-defeating beliefs) and emotional coping, positive effects on SPCS (leadership competence and policy control) and personal agency, and indirect negative effects on dominance orientation and anti-egalitarianism (divided into two dimensions each);
(2) Positive effects of psychosocial uncertainty (within work, relationships, and self-defeating beliefs) on emotional coping (a), and that both will have negative effects on personal agency (b) and SPCS (leadership competence and policy control) (c), and positive effects on dominance orientation and anti-egalitarianism (d);
(3) Positive effects of personal agency on SPCS (leadership competence and policy control) and negative effects on dominance orientation and anti-egalitarianism;
(4) Negative effects of SPCS (leadership competence and policy control) on dominance orientation and anti-egalitarianism

## Materials and Methods

### Procedures

This nonclinical community, a convenience sample, was mostly collected online between December 2017 and March 2018 by disseminating the study through various professional and informal networks and counting with the collaboration of training centres to invite trainees to participate in face-to-face data collection. The survey clarified the aims of the research, provided clear instructions, and guaranteed confidentiality and anonymity.

### Participants

The sample comprises 633 participants: 73% females; age average, 38.4 (standard deviation, 11.2); 63% employed and 37% unemployed. The participants were invited to identify their perceptions of their income in a five-point Likert scale: 9% identified at the lower end; 21% as low; 48% in the middle; 20% at an upper level; and 2% at the highest income level. In terms of schooling, 22% had up to 9 years of schooling; 15% had 12 years of schooling or a vocational course equivalent to this; and 63% had higher education, which is explained by the online data gathering strategy. Table 1 details the sample characterisation.

**TABLE 1:** Sample demographic characteristics.

	Gender		Perceived Income Level					Schooling			Level of Precarity			Age
	Male	Female	Lower (1)	(2)	Middle (3)	(4)	Upper (5)	9 Y	12 Y	HE	Permanent Workers	Precarious Workers	Unemployed	
N = 633	163 (27%)	460 (73%)	55 (9%)	128 (21%)	297 (48%)	123 (20%)	15 (2%)	144 (23%)	94 (15%)	396 (63%)	216 (34%)	178 (28%)	235 (37%)	38.4 (11.2)

*Gender, the income level, schooling, and the level of precarity characterised by n and (%); age characterised as M (SD); Y, years; HE, higher education.*

### Materials

Table 2 presents the instruments used in this study, identifying internal consistency (α) results for this sample. The Lamb-scale dimension financial access (Muller et al., 2005) will be used to frame individual financial circumstances and explore their potential impact on this model. This scale was adapted to Portuguese by Sousa-Ribeiro (2013; Sousa-Ribeiro et al., 2014).

**TABLE 2:** Instruments.

Instruments	Dimensions and internal consistency (α) for the full sample (*n* = 635)	Item Example
Financial Access Dimension from the Latent and Manifest Benefits Scale – LAMB – Scale (Muller et al., 2005; adapt. Sousa-Ribeiro, 2013)	6 items (0.93)	From the income I receive I (often/rarely) have money left for savings.
Psychosocial Uncertainty Scale (PS-US, Lucas Casanova et al., 2021)	Psychosocial consequences of uncertainty at work – 5 items (0.78); within relationships and community living – 3 items (0.70); self-defeating beliefs on coping with uncertainty – 2 items (0.67)	When I hear about unemployment rates increasing, I worry about my future
Emotional Uncertainty Dimension from the Uncertainty Response Scale (URS, Greco and Roger, 2001; adapt. Lucas Casanova et al., 2019b)	11 items (0.92)	Facing uncertainty is a nerve-wracking experience
Personal Agency Scale (created for this study, based on Fryer, 1992, 1998)	7 items; unidimensional (0.81)	My life flies before my eyes, without my being able to control it.
Social Dominance Orientation SDO-7s (Ho et al., 2015, based on the adapt. Rodrigues, 2017)	Pro-dominance – 2 items (0.50) Anti-dominance – 2 items (0.50) Pro-egalitarianism – 2 items (0.74) Anti-egalitarianism – 2 items (0.45)	Some groups of people are simply inferior to other groups.
Socio-political Control – Adults (Peterson et al., 2006), based on the Portuguese adaption of the Socio-political Control Scale for Youth (Peterson et al., 2011; adapt. Rodrigues et al., 2016)	Policy control – 7 items (0.79) Leadership – 7 items (0.85)	People like me are generally well qualified to participate in political activity and decision-making in our country.
Sociodemographic Questionnaire	Sociodemographic and professional situation characterisation variables.	

The URS and PS-US were also previously validated for Portuguese (Lucas Casanova et al., 2019b, 2021). Here, only the dimension emotional coping of the URS will be used since it proved to be the one that had more explanatory power for issues concerned with socioeconomic circumstances and its relationship with the dimensions of the PS-US in previous studies (Lucas Casanova et al., 2021).

The Personal Agency Scale was developed for this study, so exploratory factor analysis (EFA) and confirmatory factor analysis (CFA) were performed following standard procedures (Tabachnick and Fidell, 1996/2007; Brown, 2006) by randomly dividing the sample into two and using half for EFA and half for CFA.

The SPCS for Youth had already been adapted to Portuguese (Rodrigues et al., 2016). Considering the similarities between that version and the adults one, it was decided to adopt the adults version based on the translation previously performed for the youth version. Therefore, the process of validation was performed, following the procedures above.

Regarding social dominance and egalitarianism, the SDO scale (Pratto et al., 1994, 2006) had already been adapted to Portuguese (Giger et al., 2015; Rodrigues, 2017). However, the SDO7-short scale, with eight items, was used (Ho et al., 2015). Considering the original validation results, CFA was performed following the factor structure proposed by the authors. Nevertheless, the final CFA model is different from the original research (Ho et al., 2015), which is further explained in Supplementary Materials. It is worth mentioning that internal consistency results of this scale are lower, as expected since each dimension is composed of only two items (instead of four in the original version). Despite this, the reliability composite (RC) and average variance extracted (AVE) results show the potential of the version used in this study (Briggs and Cheek, 1986; HairJr., And erson et al., 1998). Moreover, for clarity purposes, and considering this version is different from the original one, we are naming the dimensions of the SDO7-S in a slightly different manner than the original authors while retaining its core meaning. Therefore, the dimensions are organised according to substantive meaning of items and their pro or anti methodological orientation in four dimensions: pro-dominance, anti-egalitarianism, anti-dominance, and pro-egalitarianism.

All the results from these processes (EFA, CFA, internal consistency) for all scales are presented in Supplementary Materials. Convergent and divergent kinds of validity are also addressed, presenting correlations between all the variables used in this study.

### Data Analysis

Missing values’ (m.v.) patterns were analysed through Little’s test (Little and Schenker, 1995). An obtained significant *p* value indicates the existence of a m.v. pattern in our data. However, only one item had 2% of m.v., and five items reached 1.4% m.v. Therefore, considering these percentages and the fact that we used two data collection methods (online and face-to-face) and that these m.v. were found only on face-to-face participants, we did not consider this problematic (Schafer, 1999; Dong and Peng, 2013). So, IBM SPSS Statistics 24 was used to perform descriptive statistics (excluding missing values (m.v.) cases’ list wise) and IBM SPSS Amos 24 for the mediation SEM (m.v. were imputed using regression imputation according to the CFA’s structure of each measure).

## Results and Discussion

After testing the factorial and measurement validity of all scales as previously mentioned, a Structural Equation Model (SEM) was performed to test if financial access has effects on psychosocial uncertainty and emotional uncertainty (expecting it to protect against it), personal agency and SPC (potentially increasing both), and on SDO; if dimensions of psychosocial uncertainty have effects on emotional coping, personal agency, SPC, and SDO, expecting psychosocial uncertainty to have greater effects than emotional coping and personal agency, possibly undermining SPC and having effects on greater levels of SDO (while personal agency could have the inverse effect), if SPC has a negative effect on SDO, and to explore which of these variables may act as mediators in this model. The definition of each effect tested was based on the literature review and previous results on the relationship between financial access, psychosocial uncertainty, and emotional coping (Lucas Casanova, 2021; Lucas Casanova et al., 2021), adding direct effects of financial access on SPCS and SDO (as suggested by the literature review). Regarding the relationship between psychosocial uncertainty, SPC and SDO, preliminary models were tested for psychosocial uncertainty and SPC, and psychosocial uncertainty and SDO. Paths were defined through theoretical guidance and considering results from a pilot study and only the significant effects were retained for this complete model. For personal agency and emotional coping, which were being explored as potential mediators, all paths with SPCS and SDO were tested. The effects of both dimensions of SPCS on all dimensions of SDO were also tested.

**FIGURE 1:**
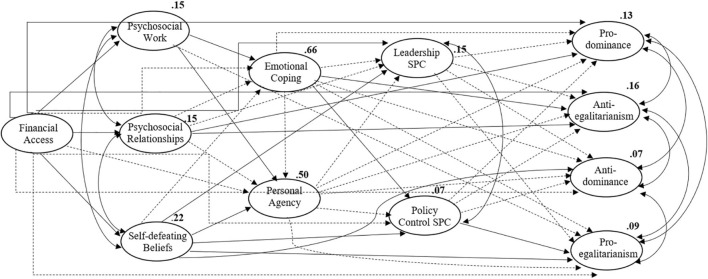
Model A, representing all paths established.

The global quality of adjustment of the model was tested with the maximum likelihood method. The indices and values of reference offered by Kline (2005) were used: Comparative Fit Index – CFI above 0.90, the root mean square error of approximation – RMSEA, P [rmsea ≤ 0.05] below 0.80; chi-square test and chi-square/degrees of freedom between 1 and 2 (Cheung and Rensvold, 2002). The model (Model A) achieved an acceptable quality of adjustment, considering the following indices: χ^2^/df = 2.13, CFI = 0.90, TLI = 0.89; RMSEA = 0.042; P [rmsea ≤ 0.05] > 0.99 (Table 3). Indeed, considering the complexity of the model and the sample size, these results could be considered as acceptable for an exploratory model (Cheung and Rensvold, 2002; Nye and Drasgow, 2011). Moreover, they may also be affected by issues with the SPCS and SDO scale, as discussed in the Supplementary Material. Figure 1 presents the model with significant and non-significant paths tested through bootstrapping with two-tailed significance. The figure also presents the explanatory power of the model for each of the dependent variables. It shows that financial access accounts for 15% of psychosocial uncertainty within work, 15% within relationships, and 22% for self-defeating beliefs. These variables account for 66% of emotional coping strategies towards uncertainty, demonstrating the relevance of financial issues and psychosocial experiences of uncertainty for coping with it. The model explains 50% of personal agency scores, which may suggest negative effects of financial deprivation and uncertainty on it. Additionally, it explains 15% of leadership competence on sociopolitical issues, 7% of policy control, 13% of pro-dominance beliefs, 16% of anti-egalitarianism beliefs, 7% of anti-dominance beliefs, and 9% of pro-egalitarianism beliefs.

**TABLE 3:** Goodness of fit indices for the mediation SEM Models A and B.

	*χ*^2^(df)	*p* value	*χ*2/df	CFI	TLI	RMSEA	LO 90	HI 90	PCLOSE	SRMR
Model A	3040 (1427)	*p* < 0.001	2.13	0.90	0.89	0.042	0.040	0.044	>0.9	0.053
Model B	3049 (1430)	*p* < 0.001	2.13	0.90	0.89	0.042	0.040	0.044	>0.9	0.054

Therefore, the model seems to support the basic assumption of the present study that levels of financial access and psychosocial uncertainty account for part of difficulties of individuals in terms of the adoption of emotional coping strategies, personal agency, impacting SPC, and SDP. Thus, those macrosocial circumstances affect experiences at the micro and meso levels. It suggests that precarious social experiences (with financial deprivation and more uncertainty in the social context) influence SPC and SDO beliefs negatively towards more social dominance and less egalitarianism. Moreover, results show that financial deprivation and uncertainty within the social context may undermine personal agency (Fryer, 1992, 1998; Marris, 1996) and affect people’s sense of SPC. These may then influence the development of beliefs in social dominance and anti-egalitarianism. Social discourses on competitiveness, meritocracy, and the Protestant work ethic instigate distrust and individualisation, which may generate a disengagement from collective action. This may lead people to seek simple political messages that appear to solve everything by blaming victims for their difficulties (Marcuse, 1964/1991; Bauman, 2001; Stiegler, 2004/2011; Coimbra and Menezes, 2009; Hogg, 2014). These processes seem to reflect socially created forms of anomie and alienation (Standing, 2011; Tenhouten, 2016) with roots in social uncertainty, which lead subordinate groups to become complicit with dominant groups *via* self-debilitation or false consciousness. Therefore, strategies used towards reducing fear, anxiety, and uncertainty (Jost et al., 2003) may undermine their psychological empowerment, working as self-defeating strategies (Zimmerman, 1995; Sidanius and Pratto, 1999; Pratto et al., 2006). However, personal agency does not act as a mediator in this model towards SPC and SDO (although it is affected by the independent variables of the model in the direction expected), which will be discussed further ahead.

**FIGURE 2:**
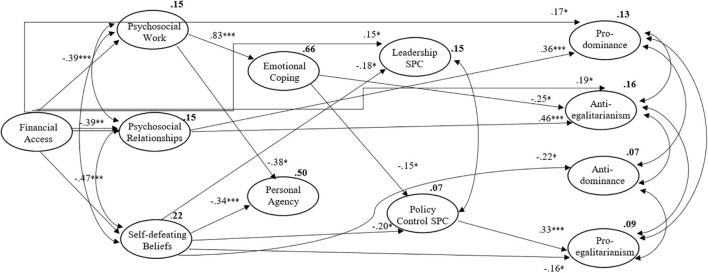
Model A, representing significant effects; ^∗∗∗^*p* < 0.001; ^∗∗^*p* < 0.005; ^∗^*p* < 0.05.

Figure 2 presents only the significant effects of the abovementioned model (all the non-significant paths were omitted but retained in the model). The first part of the model presents similar results to previous studies (Lucas Casanova, 2021; Lucas Casanova et al., 2021). It shows that financial access protects against experiencing psychosocial uncertainty within work, relationships, and developing self-defeating beliefs and that psychosocial uncertainty at work has a major effect on emotional coping, as previously found. These results provide further support for the thesis proposed by Marris (1996) that uncertainty is unequally distributed in society, as well as the power to cope with it, showing that there are social origins of uncertainty, for which financial access seems to be a major factor (Fryer, 1992, 1998). These explain the fact that people adopt self-defeating strategies to cope with uncertainty—in this case, emotional coping strategies. This is a relevant result since coping with uncertainty has been mainly studied as an intra-psychic trait-like feature, disregarding the crucial influence of environmental circumstances for developing strategies to cope with uncertainty (Lucas Casanova et al., 2019b, 2021). However, financial access does not directly affect emotional coping and personal agency but has indirect effects, which will be further discussed. Table 4 presents direct effects. All the following results are standardised betas.

**TABLE 4:** Direct effects for the mediation SEM Model A.

	Financial Access (95% CI) *p*	Psychosocial (Work) (95% CI) *p*	Psychosocial (Relationships) (95% CI) *p*	Psychosocial (SD Beliefs) (95% CI) *p*	Emotional Coping (95% CI) *p*	Personal Agency (95% CI) *p*	Leadership (SPC) (95% CI) *p*	Social Policy (95% CI) *p*
Psychosocial (Work)	**−0.39 (−0.47; −0.28) 0.002**							
Psychosocial (Relationships)	**−0.39 (−0.48; −0.28) <0.001**							
Psychosocial (SD Beliefs)	**−0.47 (−0.57; −0.38) <0.001**							
Emotional Coping	0.09 (0.007; 0.18) 0.073	**0.83 (0.66; 1.05) <0.001**	0.003 (−0.24; 0.19) 0.98	0.05 (−0.07; 0.17) 0.38				
Personal Agency	0.070 (−0.05; 0.18) 0.2	**−0.38 (−0.67; −0.10) 0.01**	**−**0.17 (−0.38; 0.06) 0.13	**−0.34 (−0.48; −0.19) <0.001**	0.04 (−0.14; 0.23) 0.70			
Leadership (SPC)	**0.15 (0.03; 0.27) 0.012**		−0.03 (−0.19; 0.12) 0.71	**−0.18 (−0.34; −0.02) 0.026**	−0.10 (−0.24; 0.05) 0.19	0.07 (−0.07; 0.23) 0.35		
Social Policy (SPC)	0.07 (−0.06; 0.20) 0.23			**−0.20 (−0.38; −0.02) 0.028**	**−0.15 (−0.27; −0.02) 0.027**	−0.09 (−0.26; 0.08) 0.30		
Pro-Dominance (SDO)	**0.17 (0.04; 0.31) 0.009**		**0.36 (0.16; 0.60) <0.001**		0.02 (−0.20; 0.16) 0.87	−0.07 (−0.23; 0.09) 0.36	0.16 (−0.04; 0.36) 0.12	−0.16 (−0.36; 0.03) 0.11
Anti-egalitarianism (SDO)	**0.19 (0.04; 0.35) 0.018**		**0.46 (0.24; 0.69) <0.001**		**−0.25 (−0.45; −0.05) 0.018**	−0.01 (−0.31; 0.07) 0.19	0.18 (−0.05; 0.43) 0.11	−0.18 (−0.42; 0.04) 0.13
Anti-Dominance (SDO)	−0.11 (−0.24; −0.02) 0.087			**−0.22 (−0.37; −0.05) 0.016**	0.07 (−0.07; 0.20) 0.31	−0.03 (−0.20; 0.14) 0.69	−0.01 (−0.24; 0.22) 0.96	0.17 (−0.04; 0.39) 0.13
Pro-egalitarianism (SDO)	−0.027 (−0.14; 0.08) 0.58	0.025 (−0.16; 0.21) 0.75		−0.16 (−0.31; 0.003) 0.053	0.06 (−0.11; 0.22) 0.51	−0.01 (−0.17; 0.18) 0.96	−0.16 (−0.35; 0.03) 0.11	**0.33 (0.16; 0.51) <0.001**

*Significance levels assessed through bootstrapping (2,000 samples) using the two-tailed significance of the bias-corrected confidence intervals for 95%; CI – 95% confidence intervals. Bold: significant effects.*

Psychosocial uncertainty within work and self-defeating beliefs in coping with uncertainty have significant negative direct effects on personal agency. However, these are non-significant for psychosocial uncertainty within relationships and for emotional coping. Results follow the expected direction, partially confirming our hypothesis on this. Hence, it seems that psychosocial uncertainty within relationships and community living (expressing experiences of distrust) are not as detrimental to personal agency as uncertainty within the work context, confirming the weight of working experiences and the labour market for personal agency (Fryer, 1998; Blustein, 2019). On the other hand, self-defeating beliefs in coping with uncertainty are intimately related with projecting into the future, explaining its role in feelings of agency towards one’s life (Marris, 1996). Also, emotional coping strategies with uncertainty do not significantly influence personal agency. Although not expected, this result provides support for the distinction between the psychological experience of social uncertainty and coping strategies towards uncertainty, demonstrating that psychosocial uncertainty may exert a greater influence than intra-psychic forms of coping with uncertainty, which was expected in the form of effect size (Marris, 1996; Lucas Casanova et al., 2019b, 2021).

Leadership competence within SPC is directly and positively influenced by financial access, while negatively influenced by self-defeating beliefs, as hypothesised. However, it is not directly affected by the other psychosocial uncertainty dimensions. Research shows that people with higher educational levels (which are strongly correlated with financial access) show higher leadership competence levels (Zimmerman and Zahniser, 1991). On the other hand, self-defeating beliefs in coping with uncertainty demonstrate the corrosive power of uncertainty, undermining personal agency, which is congruent with the decline of leadership competence as a belief in the ability of organising groups of people (Zimmerman and Zahniser, 1991; Zimmerman, 1995). Policy control is negatively influenced by emotional coping and self-defeating beliefs, as expected, although not by the other dimensions of PS-US, at least through direct effects. Thus, the adoption of emotional strategies to cope with uncertainty (which are highly correlated with anxiety – Thielsch et al., 2015), as a self-defeating strategy may lead people to disengage from sociopolitical issues due to the anxiety they create. This is congruent with analyses of alienation and anomie that reflect disbelief of people in their ability to influence policy (Standing, 2011; Tenhouten, 2016). On the other hand, it is congruent with results that show that threat affects avoiding information seeking regarding sociopolitical issues (Shepherd and Kay, 2012), and distrust created by social uncertainty may also contribute to it. Furthermore, experiences of financial deprivation and of the psychological consequences of social uncertainty may create the emotional outcomes of anomie mentioned by Tenhouten (2016), as derisiveness against others (in active forms of anomie) or shame (related to loss of status, such as the one experienced as a result of unemployment) and fear of failure (in passive forms), which are connected with the development of sociopolitical and social dominance beliefs.

Moreover, financial access has significant direct positive effects on pro-dominance and anti-egalitarianism, which is consistent with the literature that demonstrates that increased financial access may directly contribute to an increased orientation to social dominance (Sidanius and Pratto, 1999). However, in this study, we also aimed to explore potential indirect effects of financial access when mediated by psychosocial uncertainty that could demonstrate the complexity of the impact of financial access, which will be analysed further ahead. There are also significant direct positive effects of psychosocial uncertainty within relationships and community living on pro-dominance and anti-egalitarianism, showing the impact of uncertainty and distrust in community living on SDO beliefs. This suggests that, as expected, community living and relationships based on distrust, individualism, and competitiveness contribute to social dominance and anti-egalitarianism (Bauman, 2001; Coimbra and Menezes, 2009; Lucas Casanova et al., 2019a). Furthermore, empirical results show that individuals with high SDO levels show high levels of nihilism and vegetativeness, which may be related to negative perceptions or feelings of meaningless towards their relationships with others and social institutions (Nicol, 2007), which seems to support our results. However, self-defeating beliefs negatively impact anti-dominance. Thus, individuals with more self-defeating beliefs in coping with uncertainty may become less oriented to anti-social dominance and to pro-egalitarianism due to their burdened experiences with psychosocial uncertainty. These experiences may generate disbelief in their ability to cope with uncertainty, which provides support for the role of psychosocial uncertainty for SDO and egalitarianism. Indeed, psychosocial uncertainty may reflect HE legitimizing ideologies that have implications for precarity and unemployment policies, reflecting values of meritocracy, competitiveness, individualism, and the protestant work ethic (Pratto et al., 2006).

However, personal agency does not contribute to SPC and SDO, as a hypothesised mediator, which may have different possible explanations. On the one hand, one can consider that the scale that was developed does not adequately tap into the construct of personal agency, despite its psychometric results that were proved satisfactory. On the other hand, it is possible that personal agency may not be that relevant for SPC and SDO, since it is mostly related to the development of personal, family, or professional life projects and feeling in control of one’s life. Despite it being influenced by financial access and psychosocial uncertainty, its more personal dimension may not impact how people believe they can control their sociopolitical environments and influence their beliefs in SDO and egalitarianism. Emotional coping has a significant direct negative effect on anti-egalitarianism, which is the opposing direction that would be expected. We will discuss this direct effect on the next section focused on indirect effects, given its relationship with an indirect effect that was found.

Interestingly, when analysing SPCS effects on SDO dimensions, it is possible to identify different trends for each of SPCS dimensions even though only one direct effect is significant. Leadership competence has no significant direct effects, but their orientation is positive for pro-dominance and anti-egalitarianism and negative for anti-dominance and pro-egalitarianism. In contrast, policy control has a significant direct positive effect on pro-egalitarianism and the opposite orientation regarding the other dependent variables, although non-significant. These results are relevant to distinguish the importance of these two dimensions of the intrapersonal feature of psychological empowerment. As previously mentioned, empirical results show that high policy control levels are more relevant for school participation and perceived importance of school than high levels of leadership competence (Zimmerman and Zahniser, 1991). These results, along with the results of this study, show that the policy control dimension, reflecting knowledge of policy and political issues and the perception of the ability to influence them, can be a promotor of egalitarianism. At the same time, leadership competence, in contrast, may lead to pro-dominance and anti-egalitarianism. This shows that self-perceptions on the ability to organise groups of people towards sociopolitical issues do not guarantee low SDO levels, particularly in a mediation model that takes into account the impact of financial access and psychosocial uncertainty.

Table 5 presents indirect effects, showing that financial access has significant indirect effects on all variables but pro-egalitarianism in the direction expected (reducing psychosocial uncertainty and emotional coping strategies, increasing personal agency, increasing policy control, and leadership in SPC, reducing pro-dominance and anti-egalitarianism, and increasing anti-dominance beliefs). Even though financial access did not have a significant direct effect on personal agency, it has a significant positive indirect effect through psychosocial uncertainty at work and self-defeating beliefs, which have a direct effect on personal agency. This result demonstrates the connection between financial deprivation and precarious forms of work and personal agency, showing that financial access can protect personal agency when considering psychosocial uncertainty within work (Fryer, 1992, 1998). Moreover, these results, when compared with direct results, confirm the complexity of financial access effects since, when they are mediated by psychosocial uncertainty and the other variables in the model, financial access may paradoxically protect from developing social dominance and anti-egalitarianism beliefs.

**TABLE 5:** Indirect effects for the mediation SEM Model A.

	Financial Access (95% CI) *p*	Psychosocial (Work) (95% CI) *p*	Psychosocial (Relationships) (95% CI) *p*	Psychosocial (SD Beliefs) (95% CI) *p*	Emotional Coping (95% CI) *p*	Personal Agency (95% CI) *p*
Emotional Coping	**−0.34 (−0.44; −0.24) 0.002**					
Personal Agency	**0.36 (0.27; 0.46) <0.001**	0.03 (−0.12; 0.20) 0.68	0 (−0.03; 0.02) 0.99	0.00 (−0.01; 0.04) 0.41		
Leadership (SPC)	**0.15 (0.07; 0.22) 0.002**	−0.10 (−0.24; 0.02) 0.092	−0.01 (−0.07; 0.02) 0.38	−0.03 (−0.10; 0.02) 0.25	0.00 (−0.01; 0.04) 0.42	
Social Policy (SPC)	**0.09 (0.02; 0.17) 0.019**	−0.09 (−0.20; 0.01) 0.069	0.02 (−0.02; 0.08) 0.41	0.02 (−0.03; 0.10) 0.39	−0.00 (−0.05; 0.01) 0.46	
Pro-Dominance (SDO)	**−0.14 (−0.26; −0.06) 0.002**	0.01 (−0.13; 0.15) 0.88	−0.000 (−0.04; 0.05) 0.80	−0.02 (−0.04; 0.08) 0.51	0.000 (−0.03; 0.05) 0.71	0.03 (−0.00; 0.09) 0.067
Anti-egalitarianism (SDO)	**−0.14 (−0.25; −0.06) 0.002**	−0.17 (−0.35; 0.006) 0.057	0.01 (−0.07; 0.10) 0.72	−0.02 (−0.05; 0.11) 0.48	0.01 (−0.04; 0.05) 0.74	0.03 (−0.00; 0.11) 0.074
Anti-Dominance (SDO)	**0.10 (0.03; 0.18) 0.008**	0.05 (−0.06; 0.17) 0.31	0.01 (−0.03; 0.07) 0.52	0.01 (−0.09; 0.05) 0.65	−0.03 (−0.07; 0.01) 0.12	−0.02 (−0.08; 0.02) 0.37
Pro-egalitarianism (SDO)	0.06 (−0.009; 0.13) 0.094	0.04 (−0.12; 0.21) 0.63	0.01 (−0.03; 0.07) 0.45	−0.02 (−0.10; 0.05) 0.53	−0.04 (−0.09; 0.01) 0.092	−0.04 (−0.12; 0.002) 0.067

*Significance levels assessed through bootstrapping (2,000 samples) using the two-tailed significance of the bias-corrected confidence intervals for 95%; CI – 95% confidence intervals. Bold: significant effects.*

Psychosocial uncertainty at work does not have significant indirect effects on SDO dimensions, contrary to what was expected and neither does psychosocial uncertainty within relationships and self-defeating beliefs. However, in this model, psychosocial uncertainty at work, within relationships, and self-defeating beliefs have no indirect effects on the other variables of the model, as emotional coping and personal agency. Indeed, financial access seems to dominate the model, suppressing the effects of other variables. Table 6 presents the total effects found in the model.

**TABLE 6:** Total effects for the mediation SEM Model A.

	Financial Access (95% CI) *p*	Psychosocial (Work) (95% CI) *p*	Psychosocial (Relationships) (95% CI) *p*	Psychosocial (SD Beliefs) (95% CI) *p*	Emotional Coping (95% CI) *p*	Personal Agency (95% CI) *p*	Leadership (SPC) (95% CI) *p*	Social Policy (95% CI) *p*
Psychosocial (Work)	**−0.39 (−0.47; −0.28) 0.002**							
Psychosocial (Relationships)	**−0.39 (−0.48; −0.28) <0.001**							
Psychosocial (SD Beliefs)	**−0.47 (−0.57; −0.38) <0.001**							
Emotional Coping	**−0.26 (−0.35; −0.17) <0.001**	**0.83 (0.66; 1.05) <0.001**	0.00 (−0.24; 0.19) 0.98	0.05 (−0.07; 0.17) 0.38				
Personal Agency	**0.43 (0.34; 0.51) <0.001**	**−0.35 (−0.55; −0.14) 0.002**	−0.17 (−0.37; 0.05) 0.12	**−0.34 (−0.47; −0.19) <0.001**	0.04 (−0.14; 0.23) 0.70			
Leadership (SPC)	**0.30 (0.21; 0**.**38) <0.001**	−0.10 (−0.24; 0.02) 0.092	−0.04 (−0.19; 0.11) 0.60	**−0.21 (−0.34; −0.07) 0.002**	−0.09 (−0.23; 0.05) 0.21	0.07 (−0.07; 0.23) 0.35		
Social Policy (SPC)	**0.16 (0.06; 0.26) 0.002**	−0.09 (−0.20; 0.01) 0.069	0.02 (−0.02; 0.08) 0.41	**−0.18 (−0.34; −0.02) 0.03**	**−0.15 (−0.28; −0.02) 0.024**	−0.09 (−0.26; 0.08) 0.30		
Pro-Dominance (SDO)	0.03 (−0.11; 0.16) 0.65	0.01 (−0.13; 0.15) 0.88	**0.36 (0.14; 0.59) <0.001**	0.02 (−0.04; 0.08) 0.51	0.01 (−0.20; 0.16) 0.887	0.05 (−0.20; 0.11) 0.56	0.16 (−0.04; 0.36) 0.12	−0.16 (−0.36; 0.03) 0.11
Anti-egalitarianism (SDO)	0.04 (−0.09; 0.18) 0.51	−0.17 (−0.35; 0.01) 0.06	**0.47 (0.27; 0.70) <0.001**	0.02 (−0.05; 0.11) 0.48	**−0.25 (−0.45; −0.05) 0.021**	−0.10 (−0.28; 0.10) 0.33	0.18 (−0.05; 0.43) 0.11	−0.18 (−0.42; 0.04) 0.13
Anti-Dominance (SDO)	−0.01 (−0.12; 0.10) 0.80	0.05 (−0.06; 0.17) 0.31	0.01 (−0.03; 0.07) 0.52	**−0.24 (−0.38; −0.09) 0.004**	0.04 (−0.09; 0.18) 0.50	−0.05 (−0.22; 0.12) 0.54	−0.01 (−0.24; 0.22) 0.96	0.17 (−0.04; 0.39) 0.13
Pro-egalitarianism (SDO)	0.03 (−0.06; 0.13) 0.48	0.06 (−0.06; 0.18) 0.29	0.01 (−0.03; 0.07) 0.45	**−0.19 (−0.31; −0.04) 0.011**	0.02 (−0.13; 0.19) 0.73	−0.05 (−0.22; 0.13) 0.59	−0.15 (−0.35; 0.03) 0.11	**0.33 (0.16; 0.51) <0.001**

*Significance levels assessed through bootstrapping (2,000 samples) using the two-tailed significance of the bias-corrected confidence intervals for 95%; CI – 95% confidence intervals. Bold: significant effects.*

Therefore, our first hypothesis was partially confirmed *via* direct or indirect effects in the direction expected between financial access and all the model variables, except for pro-egalitarianism. Our second hypothesis was partially confirmed, showing (a) the expected effects of psychosocial uncertainty on emotional coping (mostly from psychosocial uncertainty within work, even though all were tested), and that both have negative effects on personal agency (b). Regarding their effects on SPC (c), these were confirmed for self-defeating beliefs (on both dimensions) and for emotional coping (only on social policy). Concerning their direct effects on SDO (d), these were confirmed for the PS-US. In contrast, emotional coping was only confirmed for social policy and anti-egalitarianism. Our third hypothesis was rejected, demonstrating that personal agency does not contribute as a mediator to the model in explaining SPC and SDO. And our fourth hypothesis was partially confirmed, showing effects of social policy on pro-egalitarianism.

**FIGURE 3:**
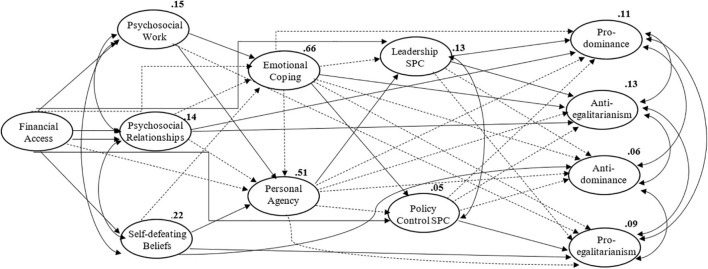
Model B, representing all paths established.

**FIGURE 4:**
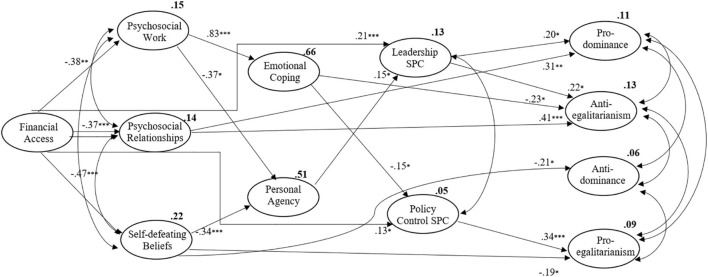
Model B, representing significant effects; ^∗∗∗^*p* < 0.001; ^∗∗^*p* < 0.005; ^∗^*p* < 0.05.

Considering results reveal more direct effects and only indirect effects from financial access, not proving our proposed mediation, it was decided to explore if psychosocial uncertainty could have indirect effects that were not being assessed due to the powerfulness of its direct effects and due to the powerfulness of financial access effects. Therefore, a new model was tested in which the direct effects of psychosocial uncertainty dimensions on SPC dimensions and the direct effects of financial access on SDO dimensions were eliminated (Model B) – Table 3 presents the global quality of adjustment of the model, which is similar since only three paths were eliminated. Figure 3 presents the model with significant and non-significant effects. Figure 4 and Table 7 present direct effects, which follow the same patterns as the previous ones, except for the direct effect of financial access on social policy, which becomes significant and higher than the indirect effect that was previously found (since it is not competing with the direct effects of psychosocial uncertainty dimensions). Personal agency has a significant positive effect on leadership competence, as was originally hypothesised. The direct effects of leadership competence on pro-dominance and anti-egalitarianism follow the same direction and become significant, showing a possible positive effect on social dominance that demonstrates how it works in the opposite orientation to social policy, which seems to have a positive effect on pro-egalitarianism.

**TABLE 7:** Direct effects for the mediation SEM Model B.

	Financial Access (95% CI) *p*	Psychosocial (Work) (95% CI) *p*	Psychosocial (Relationships) (95% CI) *p*	Psychosocial (SD Beliefs) (95% CI) *p*	Emotional Coping (95% CI) *p*	Personal Agency (95% CI) *p*	Leadership (SPC) (95% CI) *p*	Social Policy (95% CI) *p*
Psychosocial (Work)	**−0.38 (−0.47; −0.28) 0.002**							
Psychosocial (Relationships)	**−0.37 (−0.47; −0.26) <0.001**							
Psychosocial (SD Beliefs)	**−0.47 (−0.56; −0.37) <0.001**							
Emotional Coping	0.08 (−0.01; 0.18) 0.08	**0.83 (0.66; 1.06) <0.001**	−0.00 (−0.25; 0.18) 0.97	0.05 (−0.07; 0.17) 0.40				
Personal Agency	0.07 (−0.05; 0.18) 0.20	**−0.37 (−0.65; −0.08) 0.013**	−0.18 (−0.39; 0.04) 0.11	**−0.34 (−0.48; −0.19) <0.001**	0.04 (−0.14; 0.23) 0.72			
Leadership (SPC)	**0.21 (0.10; 0**.**32) <0.001**				−0.11 (−0.22; 0.01) 0.089	**0.15 (0.02; 0.28) 0.012**		
Social Policy (SPC)	**0.13 (0.002; 0.25) 0.044**				**−0.15 (−0.28; −0.02) 0.023**	−0.01 (−0.15; 0.15) 0.95		
Pro-Dominance (SDO)			**0.31 (0.11; 0.53) 0.003**		0.01 (−0.17; 0.18) 0.94	−0.02 (−0.17; 0.14) 0.849	**0.20 (−0.004; 0**.**41) 0.053**	−0.17 (−0.37; 0.03) 0.094
Anti-egalitarianism (SDO)			**0.41 (0.20; 0.63) <0.001**		**−0.23 (−0.43; −0.04) 0.022**	−0.06 (−0.24; 0.13) 0.51	**0.22 (−0.003; 0.50) 0.053**	−0.19 (−0.44; 0.04) 0.11
Anti-Dominance (SDO)				**−0.21 (−0.36; −0.04) 0.016**	0.07 (−0.06; 0.20) 0.30	−0.07 (−0.24; 0.10) 0.37	−0.03 (−0.26; 0.19) 0.82	0.19 (−0.02; 0.41) 0.08
Pro-egalitarianism (SDO)		0.008 (−0.18; 0.18) 0.91		**−0.19 (−0.32; −0.03) 0.025**	0.07 (−0.09; 0.24) 0.41	−0.04 (−0.21; 0.14) 0.67	−0.15 (−0.35; 0.02) 0.082	**0.34 (0.17; 0.50) <0.001**

*Significance levels assessed through bootstrapping (2,000 samples) using the two-tailed significance of the bias-corrected confidence intervals for 95%; CI – 95% confidence intervals. Bold: significant effects.*

This model was tested mostly to explore the impact of eliminating these paths in terms of indirect effects and further explore the relationship between the variables. Table 8 presents these indirect effects, showing that psychosocial uncertainty within work may have a significant negative indirect effect on policy control. Thus, psychosocial uncertainty within work undermines experiences of understanding the sociopolitical environment and feeling capable to influence it (policy control). There is also a significant negative indirect effect of psychosocial uncertainty within work on leadership competence. These results provide support for the eroding effect of psychosocial uncertainty within the workplace on SPC. There is also a significant (small) negative indirect effect of self-defeating beliefs in leadership competence. Therefore, psychosocial uncertainty within work seems to represent an environmental demand that undermines the intrapersonal dimension of psychological empowerment (Zimmerman and Zahniser, 1991; Zimmerman, 1995). Moreover, it supports the disempowering effect of uncertainty within work and the labour market, which may then have effects in terms of the confidence to explore, understand, and intervene in sociopolitical issues, since an individual may be burdened with everyday concerns related to survival and subsistence. On the other hand, there is a significant negative indirect effect of psychosocial uncertainty at work on anti-egalitarianism. However, this effect goes in the opposite direction, expected for the whole scale (even though it was expected since preliminary analyses had shown it in a model with PS-US and SDO). Therefore, psychosocial uncertainty at work shows an opposing function regarding the other dimensions of psychosocial uncertainty by reducing anti-egalitarianism. Although uncertainty in the social context seems to contribute to more social dominance and anti-egalitarianism beliefs, the personal experience of psychosocial uncertainty within the workplace, through precarity, unemployment, or other forms of uncertainty and insecurity at work and in the labour market, may be protective of developing anti-egalitarianism beliefs. Albeit a small indirect effect, this result seems particularly interesting in the role of personal experiences of disadvantage for developing beliefs in social dominance and egalitarianism. It is also relevant to consider it along with the significant direct negative effect of emotional coping on anti-egalitarianism since it is transported through this variable. Therefore, it seems that the psychosocial experience of uncertainty at work and emotional coping strategies towards uncertainty may lead people to become more empathic with others in similar situations, reducing anti-egalitarianism beliefs. Finally, personal agency shows a small significant indirect effect on pro-dominance, which confirms that it does not contribute greatly to the model, since personal agency experiences may not help differentiate SPC or social dominance beliefs. Table 9 presents total effects for Model B.

**TABLE 8:** Indirect effects for the mediation SEM Model B.

	Financial Access (95% CI) *p*	Psychosocial (Work) (95% CI) *p*	Psychosocial (Relationships) (95% CI) *p*	Psychosocial (SD Beliefs) (95% CI) *p*	Emotional Coping (95% CI) *p*	Personal Agency (95% CI) *p*
Emotional Coping	**−0.34 (−0.43; −0.24) 0.002**					
Personal Agency	**0.36 (0.27; 0.46) <0.001**	0.03 (−0.12; 0.21) 0.70	0 (−0.04; 0.019) 0.81	0 (−0.006; 0.039) 0.42		
Leadership (SPC)	**0.09 (0.04; 0.16) <0.001**	**−0.14 (−0.24; −0.051) 0.004**	−0.03 (−0.087; 0.019) 0.20	**−0.06 (−0.12; −0.01) 0.011**	0.01 (−0.021; 0.044) 0.57	
Social Policy (SPC)	0.04 (−0.027; 0.093) 0.22	**−0.12 (−0.23; −0.029) 0.013**	0.00 (−0.038; 0.065) 0.86	−0.01 (−0.059; 0.049) 0.83	0.00 (−0.021; 0.015) 0.85	
Pro-Dominance (SDO)	**−0.09 (−0.20; −0.01) 0.017**	0.00 (−0.13; 0.14) 0.97	0.00 (−0.049; 0.03) 0.79	−0.01 (−0.063; 0.044) 0.85	0.00 (−0.03; 0.041) 0.81	**0.03 (0.002; 0**.**092) 0.04**
Anti-egalitarianism (SDO)	**−0.08 (−0.17; −0.01) 0.025**	**−0.18 (−0.36; −0.012) 0.035**	0.01 (−0.051; 0.084) 0.82	0.00 (−0.077; 0.06) 0.91	0.00 (−0.035; 0.045) 0.83	0.03 (0.001; 0.11) 0.038
Anti-Dominance (SDO)	**0.07 (0.011; 0.14) 0.025**	0.06 (−0.037; 0.18) 0.19	0.01 (−0.016; 0.075) 0.28	0.03 (−0.025; 0.10) 0.24	−0.03 (−0.077; 0) 0.05	−0.01 (−0.061; 0.034) 0.74
Pro-egalitarianism (SDO)	0.06 (−0.002; 0.12) 0.065	0.05 (−0.092; 0.23) 0.45	0.01 (−0.018; 0.067) 0.35	0.02 (−0.032; 0.093) 0.40	−0.04 (−0.09; 0.006) 0.089	−0.02 (−0.088; 0.019) 0.24

*Significance levels assessed through bootstrapping (2,000 samples) using the two-tailed significance of the bias-corrected confidence intervals for 95%; CI – 95% confidence intervals. Bold: significant effects.*

**TABLE 9:** Total effects for the mediation SEM Model B.

	Financial Access (95% CI) *p*	Psychosocial (Work) (95% CI) *p*	Psychosocial (Relationships) (95% CI) *p*	Psychosocial (SD Beliefs) (95% CI) *p*	Emotional Coping (95% CI) *p*	Personal Agency (95% CI) *p*	Leadership (SPC) (95% CI) *p*	Social Policy (95% CI) *p*
Psychosocial (Work)	**−0.38 (−0.47; −0.28) 0.002**							
Psychosocial (Relationships)	**−0.37 (−0.47; −0.26) <0.001**							
Psychosocial (SD Beliefs)	**−0.47 (−0.56; −0.37) <0.001**							
Emotional Coping	**−0.26 (−0.35; −0.17) <0.001**	**0.83 (0.66; 1.06) <0.001**	−0.00 (−0.25; 0.18) 0.97	0.05 (−0.068; 0.17) 0.40				
Personal Agency	**0.43 (0.34; 0.52) <0.001**	**−0.33 (−0.54; −0.12) 0.002**	−0.18 (−0.39; 0.032) 0.089	**−0.34 (−0.48; −0.19) <0.001**	0.04 (−0.14; 0.23) 0.72			
Leadership (SPC)	**0.30 (0.21; 0.39) <0.001**	**−0.14 (−0.24; −0.051) 0.004**	−0.03 (−0.087; 0.019) 0.20	**−0.06 (−0.12; −0.01) 0.011**	−0.11 (−0.22; 0.024) 0.12	**0.15 (0.022; 0.28) 0.012**		
Social Policy (SPC)	**0.16 (0.06; 0.26) 0.002**	**−0.12 (−0.23; −0.029) 0.013**	0.00 (−0.038; 0.065) 0.86	−0.01 (−0.059; 0.049) 0.83	**−0.15 (−0.28; −0.017) 0.025**	−0.01 (−0.15; 0.15) 0.95		
Pro-Dominance (SDO)	**−0.09 (−0.20; −0.013) 0.017**	0.00 (−0.13; 0.14) 0.97	**0.31 (0.10; 0.53) 0.003**	−0.01 (−0.063; 0.044) 0.85	0.01 (−0.17; 0.18) 0.91	0.02 (−0.13; 0.18) 0.80	**0.20 (−0.004; 0.41) 0.053**	−0.17 (−0.37; 0.033) 0.094
Anti-egalitarianism (SDO)	**−0.08 (−0.17; −0.014) 0.025**	**−0.18 (−0.36; −0.012) 0.035**	**0.42 (0.21; 0.63) <0.001**	0.00 (−0.077; 0.06) 0.91	**−0.23 (−0.42; −0.04) 0.023**	−0.03 (−0.20; 0.16) 0.80	**0.22 (−0.003; 0.50) 0.053**	−0.19 (−0.44; 0.042) 0.11
Anti-Dominance (SDO)	**0.07 (0.011; 0.14) 0.025**	0.06 (−0.037; 0.18) 0.19	0.01 (−0.016; 0.075) 0.28	**−0.18 (−0.31; −0.037) 0.02**	0.04 (−0.087; 0.17) 0.50	−0.08 (−0.24; 0.086) 0.33	−0.03 (−0.26; 0.19) 0.82	0.19 (−0.019; 0.41) 0.08
Pro-egalitarianism (SDO)	0.06 (−0.002; 0.12) 0.065	0.06 (−0.045; 0.18) 0.26	0.01 (−0.018; 0.067) 0.35	**−0.17 (−0.27; −0.039) 0.016**	0.04 (−0.13; 0.20) 0.65	−0.06 (−0.23; 0.10) 0.44	−0.15 (−0.35; 0.017) 0.082	**0.34 (0.17; 0.50) <0.001**

*Significance levels assessed through bootstrapping (2,000 samples) using the two-tailed significance of the bias-corrected confidence intervals for 95%; CI – 95% confidence intervals. Bold: significant effects.*

## Conclusion

The finding that financial access of individuals has broad effects on this model seems to endorse that there is an impact of inequality on the psychological experience of socially created uncertainty and on sociopolitical issues, potentially reinforcing extremist and populist views. Although accounting only for a small percentage of SPC and SDO, the model seems to support this undesirable potential of financial deprivation and psychosocial uncertainty in their relationship with sociopolitical issues. The danger lies mostly in adopting HE-legitimizing myths by subordinate groups, which reinforce the power of these myths through consensus (Pratto et al., 2006). Therefore, results seem to suggest that financial deprivation and psychosocial uncertainty may contribute to limited sociopolitical analyses and limited critical consciousness (Diemer and Blustein, 2006), which may help explain recent increases in extremist and populist political worldviews. However, the experience of uncertainty at work may have the opposite effect, generating empathy towards other subordinate groups. On the other hand, it seems to lend support to the relevance of focusing on external social uncertainty rather than internal forms of uncertainty and on the role of uncertainty within work and the labour market, when researching SPC, other dimensions of psychological empowerment, and SDO. These relationships had not yet been tested, and, so, it could be useful to further study the potential impact of financial deprivation and psychosocial uncertainty on SDO and SPC.

In its connection with practice, there are implications of results for ways in which we conceive of the good society and social justice, as well as dominant psychological intervention, namely in vocational/professional counselling. Research results on these issues should be used to devise strategies and interventions that do not reproduce discourses of responsabilization through individualized forms of psychological empowerment, but foster individual and collective empowerment, acknowledging forms of social disempowerment of vulnerable groups. Thus, as Kieffer (1984) proposed, developing participatory competence entails focusing not only on perceived competence and self-efficacy (which would result in an individualised, psychologised strategy). It entails focusing on a critical understanding of the sociopolitical context, on socially created forms of uncertainty, and on the development of personal and collective resources for political action. So, fostering critical consciousness and understanding feelings of disempowerment, namely through conscientisation (Freire, 1970/1972), is crucial in the vocational/career counselling field, whether with adolescents or adults. Within these interventions, it is crucial to consider the impact of the labour market and work experiences in circumstances of inequality, disadvantage, or deprivation. This would allow people to understand the impact of sociopolitical and economic structures in their individual lives, understanding the connection between the private and the political, and not just promoting the adaptation to a particular labor market and society (Prilleltensky, 1994; Diemer and Blustein, 2006; Prilleltensky and Stead, 2012). Likewise, intervention in issues of social generativity through educational programs could prove useful to promote social responsibility, mutuality, empathy, commitment to the community, and reflection on universal values (as egalitarianism), crucial for the development of inclusive attitudes and political involvement (Morselli and Passini, 2015). Thus, learning to take care of each other (Stiegler, 2004/2011).

Regarding the limitations of this study, the fact that this is not a representative sample must be acknowledged, and future research should seek to replicate these results with a representative sample. On another note, it would be relevant to explore the impact of financial access and psychosocial uncertainty on other psychological empowerment dimensions. Moreover, this study focuses on these specific variables, although other factors could influence these results, and, so, further research would be important to examine other potential contributors to increased SDO and decreased SPC. It would also be crucial to study how this model would behave in longitudinal studies to explore potential changes in SPC and SDO according to different macrosocial moments and so test the predictive capacity of this theoretical model and confirm causal relationships. Different levels of financial access and psychosocial uncertainty may influence participants. This would be most useful if it were possible to use data to accompany major historical and social changes.

This study sought to contribute to shed light on the role of socioeconomic circumstances, psychosocial uncertainty, and the intrapersonal component of psychological empowerment for the increase of extremist, populist political views during the last years in Western countries, possibly as a result of social instability and uncertainty.

## Data Availability Statement

The raw data supporting the conclusion of this article will be made available by the authors, without undue reservation.

## Ethics Statement

The studies involving human participants were reviewed and approved by the Ethics Committee of the Faculty of Psychology and Education Sciences of the University of Porto, Portugal (Reference 2017/4-2). The participants provided their written informed consent to participate in this study.

## Author Contributions

MLC contributed for the conception and the design of the study, organisation of the database, statistical analyses, interpretation of data, and wrote the first draft of the manuscript. PC contributed for the design of the study and supervised data analyses. All authors supervised development of work, interpretation of data, contributed to manuscript revision, read, and approved the submitted version.

## Conflict of Interest

The authors declare that the research was conducted in the absence of any commercial or financial relationships that could be construed as a potential conflict of interest.

## Publisher’s Note

All claims expressed in this article are solely those of the authors and do not necessarily represent those of their affiliated organizations, or those of the publisher, the editors and the reviewers. Any product that may be evaluated in this article, or claim that may be made by its manufacturer, is not guaranteed or endorsed by the publisher.
