# Supplementary material for: Neoliberal Economic Policies’ Effects on Perceptions of Social Justice and Sociopolitical Participation in Portugal
Source: Front Psychol. 2021 Nov 29;12:694270. doi: 10.3389/fpsyg.2021.694270 (PMC8666415; doi:10.3389/fpsyg.2021.694270)
Supplement: Supplementary file 2 [file Data_Sheet_1.pdf]

Supplementary Material

Scales' validity studies

Exploratory factor analysis (EFA) and confirmatory factor analysis (CFA) were performed for the Personal Agency Scale and SPCS, by randomly dividing the sample Time 0 into two and using half for EFA and half for CFA. This sample is similar to the full sample, with 71% females; age average 37.7 (standard deviation 10.8); 22% with up to nine years of schooling, 14% with up to 12 years of schooling, and 64% with Higher Education. In terms of perceived income level, 8% consider themselves to be at the lower level, 21% at a low level, 47% at a middle level, 21% at an upper level, and 3% at an upper level.

After the EFA, all items were retained for Personal Agency. However, three items were eliminated from SPCS. Two of these were eliminated for loading below .30 (4. I like trying new things that are challenging to me; 13. I like to work on solving a problem myself rather than wait and see if someone else will deal with it), which would compromise the structural validity and the reliability of the scale, lowering Cronbach alphas.

On the other hand, it could be argued that these items are more related to the construct of openness to experience (4) and problem solving (13) than to leadership competence. Another item was eliminated for loading within the two dimensions (14. People like me are generally well qualified to participate in political activity and decision making in our country). Indeed, this item was originally part of the factor "Policy control" but loaded similarly in the two dimensions, probably because it entails a personal assessment of one's qualifications to participate in political activities and decision-making, and so, to personal feelings of leadership competence. This could also be related to the low average variance extracted that was found in

the dimension Policy control. However, the elimination of these items seems to offer a shorter version of the scale, while retaining the core construct and the scale's internal consistency. Results for both these scales' EFA's are presented in table 1.

Table 2 presents results for all the CFA's performed. CFA with Personal Agency demonstrated two items had loadings below .3 and, considering this was the first process of validation of this new scale, these were eliminated (Model A). The final CFA corresponds to Model B. The items eliminated seemed not to fully reflect the construct here assessed since they focused more on the orientation of daily issues, which may be less influenced by financial deprivation. Therefore, the items retained are focused on agency experiences that may be undermined by financial deprivation (family and life projects; planning for the future; and feelings of uncontrollability towards life, family, and career).

Considering results and modification indices for the CFA Model A of SPCS, it was decided to add two correlations between errors of the dimension SPC that share meaning (between 1. A good many local elections are important to vote in, and 16. It makes a difference who I vote for because whoever gets elected will represent my interests; and between 3. People like me are generally well qualified to participate in political activity and decision making in our country, and 8. feel like I have a pretty good understanding of the important political issues which confront our society), and one correlation between errors in the dimension Leadership competence (between 7. I would prefer to be a leader rather than a follower, and 11. I would rather have a leadership role when I'm involved in a group project) The final CFA results for SPCS correspond to Model B.

Only CFA was performed for SDO since the SDO 7 scale had already been adapted to Portuguese and the SDO7-short scale uses 8 of the 16 items of SDO7-s (Ho et al., 2015). For

consistency purposes with the validation of the other scales, the same sample was used. Firstly, the CFA model as proposed by the authors (Ho et al., 2015) was tested.

The Coefficient Cronbach Alpha, composite reliability (CR) and average variance extracted (AVE) were calculated for all scales (Table 3 and 4). Considering the very poor results for SDO7-s (Table 4), it was decided to test another CFA with the substantive and methodological (pro/anti items) dimensions all separated in four dimensions, since it seemed to better fit the data and the meaning of the constructs that underlie the scale, not repeating items per dimension. Even though this CFA does not follow the original validation proposed by Ho and colleagues (2015), and the fact that Cronbach Alphas expectedly diminish since these dimensions are composed of only two items, this version was considered to provide improved psychometric validity for the analyses intended for this study.

Thus, the final results for SDO7-s CFA correspond to Model B presented in table 3. To better illustrate this decision, table 4 presents the results of Cronbach Alpha, CR and AVE from CFA Model A, demonstrating that despite the increase in Cronbach Alpha's due to the increase in items per dimension, CR and AVE are lower in some dimensions and less consistent than in the factor structure we decided for.

The Psychosocial Uncertainty Scale has been validated to Portuguese and the process of its creation and validation can be consulted in Lucas Casanova and colleagues (2021). Its factors' Cronbach alpha, composite reliability (CR) and average variance extracted (AVE) are presented for this sample (Table 3).

The Latent and Manifest Benefits Scale - Lamb-scale (Muller et al., 2005) has been adapted to Portuguese by Sousa-Ribeiro (2013; Sousa-Ribeiro et al., 2014) and here one of its dimensions will be used – Financial access. Its internal consistency results are also presented for this sample (Table 3).

The Uncertainty Response Scale (Greco & Roger, 2001) was adapted to Portuguese by Lucas Casanova and colleagues (2019b). Here, one of its dimensions will be used: Emotional Uncertainty and its internal consistency results are presented in table 3 below.

Confirmatory Factor Analysis was performed for all these scales with the present sample – table 5.

Analyzing construct reliability and validity results for all the scales, it is possible to identify concerning issues mainly in the SPCS. Regarding the low values of AVE for policy control, it must be considered and lead to cautious interpretations of results. In what concerns the AVE values of the other scales, we consider these can be considered acceptable, since AVE is often too rigorous. The composite reliability also offers support for reliability (Malhotra & Dash, 2011). Moreover, concerns have already been expressed regarding the SDO scale, which led us to adopt another version, which provided more acceptable results.

Table 6 presents Pearson correlations between all the variables used in this study to explore their relationships and discriminant validity of the variables used by comparing them to the square root of the AVE of each variable. Results support the discriminant validity between all dimensions, except for the relationship between the two subscales of the SPCS and between the subscales of the SDO. Indeed, these results demonstrate a need to further develop both these scales, which was already expected from CR and AVE results.

Table 1.

Exploratory Factor Analysis

			**Unrotated Solution**				**Rotated Solution**		
**Scale**	**N0. Items**	**Method**	**KMO**	**Scree test analysis**	**VE**	**Rotation**	**KMO**	**Retention criteria**	**No. Items**
Personal Agency	9	PAF	.87	1 factor	1 factor= 35.6%; 2 factors=6.81%	NA (1 factor)	.87	.3	9
SPCS	17	PAF	.87	2 factors	1 factor= 31.4%;<br><br>2 factors=6.9%; 3 factors=5.3%	Direct Oblimin (2 factors)	.86	.3	14

Note: PAF – Principal Axis Factoring; KMO – Kaiser-Meyer-Olkin; VE – Variance Extracted

Table 2.

Goodness of Fit Indices – Confirmatory factor analysis for Personal Agency, SPCS, and SDO 7-s

**Scales**	**χ 2(df)**	**p value**	**χ2/ df**	**CFI**	**TLI**	**RMSEA**	LO 90	HI 90	PCLOSE
Personal Agency Model A	124 (27)	p< .001	4.60	.86	.82	.107	.088	.127	p< .001
Personal Agency Model B	25 (14)	.03	1.83	.98	.97	.052	.016	.083	.43
SPCS Model A	401 (76)	p< .001	5.31	.80	.76	.117	.106	.128	p< .001
SPCS Model B	218 (73)	p< .001	3.00	.91	.89	.080	.068	.092	p< .001
SDO Model A	20 (14)	.11	1.47	.99	.98	.039	.000	.072	.67
SDO Model B	15 (14)	.34	1.12	.997	.99	.019	.000	.060	.88

Table 3

Construct Reliability and validity for all the scales

	Full sample		CFA sample (randomly extracted from the full sample)		
**Dimensions**	**N. º Items**	**α (n=635)**	**α (n=314)**	**CR**	**AVE**
Financial Access (dimension from the Lamb-scale)	6	.93	.93	.94	.71
Psychosocial consequences at work (PS-US)	5	.78	.77	.77	.41
Psychosocial consequences relationships/communities (PS-US)	3	.70	.70	.70	.44
Self-defeating Beliefs (PS-US)	2	.67	.65	.65	.48
Emotional Coping (Dimension from the URS)	11	.92	.92	.92	.51

Personal Agency (unidimensional)	7	.81	.71	.84	.53
Policy control (SPCS)	7	.79	.79	.76	.32
Leadership (SPCS)	7	.85	.84	.84	.43
Pro-egalitarianism (SDO) – Model B	2	.74	.76	.77	.63
Anti- egalitarianism (SDO) – Model B	2	.45	.49	.49	.32
Pro-dominance (SDO) – Model B	2	.50	.52	.58	.43
Anti-dominance (SDO) – Model B	2	.50	.54	.61	.46

Note: α: Coefficient Cronbach Alpha; CR: composite reliability; AVE=average variance extracted.

Table 4

Construct Reliability and validity for SDO7-s according to CFA Model A

		Full sample	CFA sample (randomly extracted from the full sample)		
**Dimensions**	**N. º Items**	**α (n=635)**	**α (n=314)**	**CR**	**AVE**
**CFA Model A**					
SDO-E	4	.56	.57	.33	.12
SDO-D	4	.52	.53	.22	.11
Pro-trait (SDO)	4	.62	.64	.52	.22
Con-trait (SDO)	4	.71	.74	.79	.49

Note: α: Coefficient Cronbach Alpha; CR: composite reliability; AVE=average variance extracted.

Table 5.

Goodness of Fit Indices – Confirmatory factor analysis for Psychosocial Uncertainty Scale, Lamb scale (complete scale), and Uncertainty Response Scale (complete scale)

**Scales**	**χ 2(df)**	**p value**	**χ2/ df**	**CFI**	**TLI**	**RMSEA**	LO 90	HI 90	PCLOSE
Psychosocial Uncertainty Scale	72 (31)	p< .001	2.35	.95	.93	.066	.046	.085	.088
Lamb scale	1140 (508)	p< .001	2.45	.91	.90	.063	.058	.068	p< .001
Uncertainty Response Scale	581 (270)	p< .001	2.15	.92	.91	.061	.054	.067	p< .01

Table 6.

Pearson Correlations between all the latent variables in the model

		Financial Access (dimension from the Lamb-scale)	Policy Control (SPCS)	Leadership Competence (SPCS)	Anti-dominance (SDO)	Anti-egalitarianism (SDO)	Pro-dominance (SDO)	Pro-egalitarianism (SDO)	Personal Agency	Self-defeating Beliefs (PS-US)	Psychosocial consequences relationships/communities (PS-US)	Psychosocial consequences at work (PS-US)
Financial Access (dimension from the Lamb-scale)	R	1	.177**	.259**	.002	.03	.033	.018	.382**	-.403**	-.346**	-.377**
	p		< .001	< .001	.953	.457	.403	.644	< .001	< .001	< .001	< .001
Policy Control (SPCS)	R	.177**	1	.777**	.161**	-.079*	-.052	.177**	.126**	-.215**	-.159**	-.156**
	p	< .001		< .001	< .001	0.048	0.19	< .001	0.001	< .001	< .001	< .001
Leadership Competence (SPCS)	R	.259**	.777**	1	.097*	-.019	.001	.101*	.226**	-.261**	-.207**	-.215**

	p	< .001	< .001		0.014	0.629	0.99	0.011	< .001	< .001	< .001	< .001
Anti-dominance (SDO)	R	.002	.161**	.097*	1	-.705**	-.547**	.945**	.027	-.077	-.024	-.009
	p	.953	< .001	.014		< .001	< .001	< .001	.492	.053	.54	.828
Anti-egalitarianism (SDO)	R	.03	-.079*	-.019	-.705**	1	.955**	-.617**	-.092*	-.034	.167**	.101*
	p	.457	.048	.629	< .001		< .001	< .001	.02	.387	< .001	.011
Pro-dominance (SDO)	R	.033	-.052	.001	-.547**	.955**	1	-.463**	-.100*	-.054	.189**	.118**
	p	.403	.19	.99	< .001	< .001		< .001	.012	.174	< .001	.003
Pro-egalitarianism (SDO)	R	.018	.177**	.101*	.945**	-.617**	-.463**	1	.018	-.083*	-.01	.006
	p	.644	< .001	.011	< .001	< .001	< .001		.657	.036	.794	.887

Personal Agency	R	.382**	.126**	.226**	.027	-.092*	-.100*	.018	1	-.421**	-.459**	-.523**
	p	< .001	0.001	p< .001	.492	.02	.012	.657		< .001	< .001	< .001
Self-defeating Beliefs (PS-US)	R	-.403**	-.215**	-.261**	-.077	-.034	-.054	-.083*	-.421**	1	.225**	.411**
	p	< .001	< .001	< .001	.053	.387	.174	.036	< .001		< .001	< .001
Psychosocial consequences relationships/communities (PS-US)	R	-.346**	-.159**	-.207**	-.024	.167**	.189**	-.01	-.459**	.225**	1	.878**
	p	< .001	< .001	< .001	.54	< .001	< .001	.794	< .001	< .001		< .001
Psychosocial consequences at work (PS-US)	R	-.377**	-.156**	-.215**	-.009	.101*	.118**	.006	-.523**	.411**	.878**	1
	p	< .001	< .001	< .001	.828	.011	.003	.887	< .001	< .001	< .001	
Emotional Coping	R	-.237**	-.176**	-.212**	.006	.074	.107**	.014	-.414**	.304**	.608**	.701**

(Dimension from the URS)												
	p	< .001	< .001	< .001	.881	.063	.007	.733	< .001	< .001	< .001	< .001

Note. R Pearson Correlation; p value
